# Radiographic Assessment of Glenoid Morphology and Its Association with Proximal Humeral Fracture Configuration

**DOI:** 10.3390/jcm15156008

**Published:** 2026-08-02

**Authors:** İhsaniye Süer Doğan, Ahmet Çulcu, Emrah Çalışkan, Batuhan Gencer, Özgür Doğan

**Affiliations:** 1 Department of Radiology, Ankara Etlik City Hospital, Ankara 06170, Turkey; dr.ihsaniye@gmail.com; 2 Orthopaedics and Traumatology Clinic, Sadıka Sabancı State Hospital, Sakarya 54580, Turkey; dr.ahmetculcu@gmail.com; 3 Department of Orthopaedics and Traumatology, Faculty of Medicine, Koç University, Istanbul 34450, Turkey; dremrahcaliskan@gmail.com; 4 Department of Orthopaedics and Traumatology, Marmara University Pendik Training and Research Hospital, Istanbul 34899, Turkey; 5 Department of Orthopaedics and Traumatology, Ankara Bilkent City Hospital, Ankara 06800, Turkey; dr.ozgurdogan@gmail.com

**Keywords:** AO/OTA classification, fracture configuration, glenoid morphology, Neer classification, proximal humeral fracture, radiographic assessment

## Abstract

**Background:** The aim of this study was to investigate the association between glenoid anatomy and proximal humeral fracture geometry and to identify radiographic parameters associated with multi-segment and articular fractures. **Methods:** In this retrospective observational study, 113 patients treated for proximal humeral fractures between 2016 and 2019, regardless of treatment modality, were included. Demographic characteristics, injury mechanisms, and fracture classifications were recorded. Fracture geometry and complexity were evaluated using the Neer and AO/OTA classification systems. Glenoid anatomy was assessed by measuring the Critical Shoulder Angle (CSA), glenoid inclination, and glenoid version on radiographs. Multivariable ordinal logistic regression was performed to adjust for age, sex, and injury mechanism. **Results:** Older age was correlated with higher Neer and AO/OTA types (*p* = 0.031, r = 0.176; *p* = 0.013, r = 0.208, respectively). Glenoid inclination was correlated with fracture geometry and fragmentation according to the Neer classification (*p* = 0.007, r = 0.228), while glenoid inclination and version were correlated with the AO/OTA classification (*p* = 0.028, r = 0.181; *p* = 0.014, r = −0.207, respectively). However, these associations were not independently associated with fracture configuration after multivariable adjustment, whereas older age remained independently associated with higher AO/OTA classification. **Conclusions:** Glenoid morphometric parameters showed weak univariate associations with proximal humeral fracture configuration but were not independent predictors after adjustment for age, sex, and injury mechanism. These findings suggest that glenoid morphology is only one of several factors contributing to fracture configuration and warrants further investigation in larger prospective studies.

## 1. Introduction

The anatomical parameters of the glenoid have been extensively studied and its relationship with many pathologies such as rotator cuff tears, impingement syndrome, shoulder instability, and frozen shoulder has been investigated in the literature [1,2,3,4,5,6,7]. Moreover, glenoid morphology is highly associated with shoulder arthroplasty outcomes [4,5,8]. A recent study has revealed that there is a significant relationship between acetabular anatomy and proximal femur fracture types [9]. Considering the anatomical and functional similarities between the joints, as both are ball-and-socket type joints, the aforementioned study raises the question of whether the anatomical features of the glenoid are also effective on proximal humeral fracture types.

Four major segments of the proximal humerus, which are the articular surface, humeral shaft, and greater and lesser tuberosities, are key points in defining proximal humeral fracture geometry, and the Neer Classification, which has been in use since its definition in the 1970s, is based on these segments [10]. Several more parameters are effective on fracture geometry, such as the energy of the injury, the position of the arm at the time of injury, and bone quality [10,11,12]. On the other hand, to the best of our knowledge, there is no study investigating the relationship between glenoid anatomy and proximal humeral fracture geometry.

Our objective was to investigate the relationship between glenoid anatomy and proximal humeral fracture geometry and to determine the anatomical features that pose a risk for multi-segment or joint-related fractures.

## 2. Materials and Methods

After institutional review board approval (E-19-2688), all patients treated for isolated proximal humeral fractures in the study hospital between 2016 and 2019 were examined retrospectively, regardless of the preferred treatment modality. Patients with co-existing clavicle or scapula fractures and patients who have a history of previous shoulder surgery were excluded from the study. Furthermore, even though standard direct shoulder radiography and computed tomography (CT) images are requested from all patients who apply to the study hospital’s emergency department with a preliminary diagnosis of proximal humeral fracture, 39 patients (mean age: 63.3 years; Range: 19–82 years) whose shoulder radiography or CT images could not be obtained for any reason, were also excluded from the study. Considering the inclusion and exclusion criteria, direct radiography and CT images of 113 patients (mean age: 55.4 years; Range: 19–93 years) were evaluated. All evaluations were made in accordance with the Declaration of Helsinki.

Injury mechanisms, fracture type, and demographic data of the patients were recorded. Injury mechanisms were classified as low- and high-energy falls or direct trauma, based on the information obtained from the patient file. To evaluate fracture geometry, quantity, and quality of fragmentation and the relationship between fracture and joint line, Neer and AO/OTA classifications were used. To evaluate the quantity and quality of fragmentation of proximal humeral fracture, Neer Classification was preferred considering its prognostic importance and the fact that it is based on the displacement of four major segments [1]. To evaluate the articular surface involvement, AO/OTA Classification was preferred because it is based on the relationship between the fracture and the joint line (extra-articular, partial articular, or complete articular) [13].

**Figure 1:**
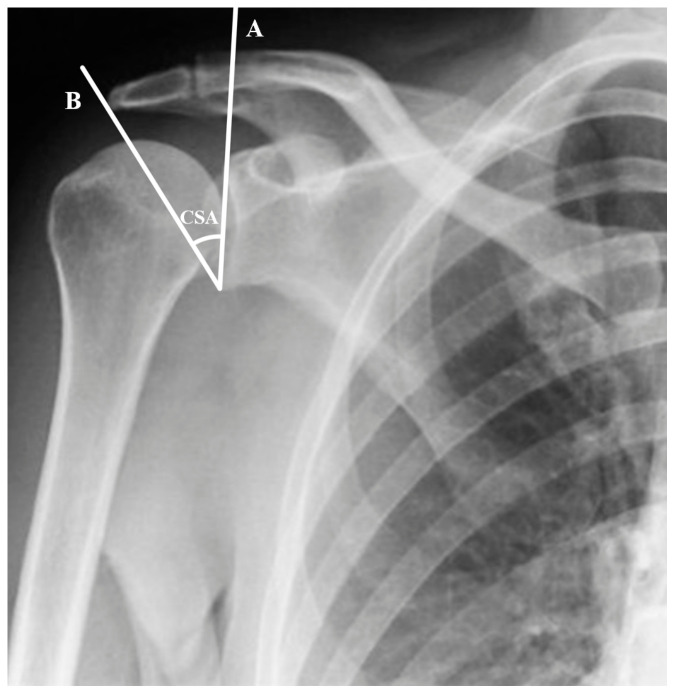
Anteroposterior shoulder radiograph demonstrating the measurement of the Critical Shoulder Angle (CSA). Three anatomical reference points are identified: the most superior point of the glenoid; the most inferior point of the glenoid and the most lateral point of the acromion. The CSA is defined as line (A) connecting the most superior and inferior points of the glenoid and the line (B) drawn from the most inferior point of the glenoid to the most lateral point of the acromion.

**Figure 2:**
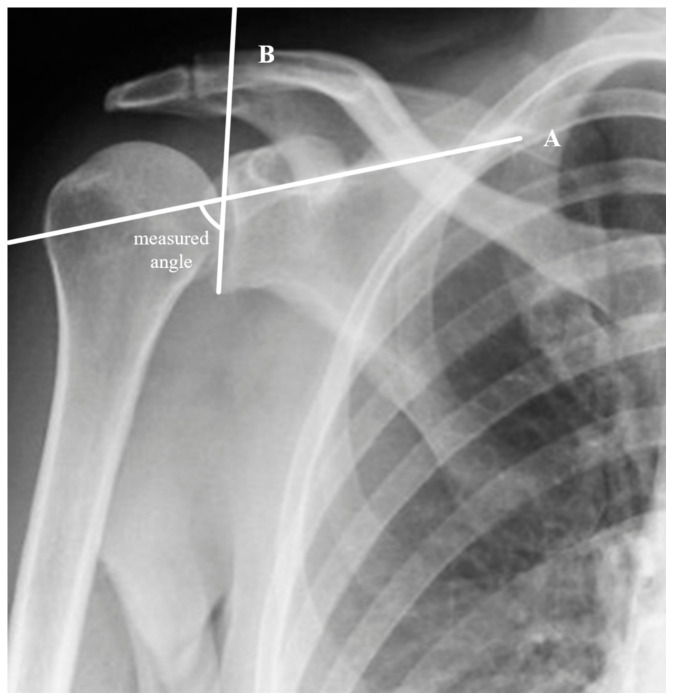
Anteroposterior shoulder radiograph demonstrating the measurement of the Glenoid inclination. Three anatomical reference points are identified: the most superior point of the glenoid; the most inferior point of the glenoid and the supraspinatus fossa. Glenoid inclination was calculated using the angle between the line (B) drawn along the supraspinatus fossa and the line (A) connecting the most superior and inferior points of the glenoid on the anterior–posterior shoulder radiograph. The inclination value was determined as the value obtained by subtracting the measured angle from 90°.

Critical Shoulder Angle (CSA), glenoid inclination, and glenoid version measurements were conducted to evaluate glenoid anatomy. All glenoid morphometric measurements were performed on the preoperative CT scans of the affected shoulder before any surgical intervention. Given that the study included proximal humeral fractures without glenoid involvement, and all the scapula fractures were excluded, the native glenoid anatomy was preserved and could be evaluated reliably. CSA, which can be measured on an anterior–posterior shoulder radiograph, is defined as the angle between the line connecting the most superior and inferior points of the glenoid and the line drawn from the most inferior point of the glenoid to the most lateral point of the acromion (Figure 1) [14,15]. Glenoid inclination was calculated using the angle between the line drawn along the supraspinatus fossa and the line connecting the most superior and inferior points of the glenoid on the anterior–posterior shoulder radiograph (Figure 2). Glenoid inclination (GI) was determined as the value obtained by subtracting the angle from 90 degrees [15,16].

**Figure 3:**
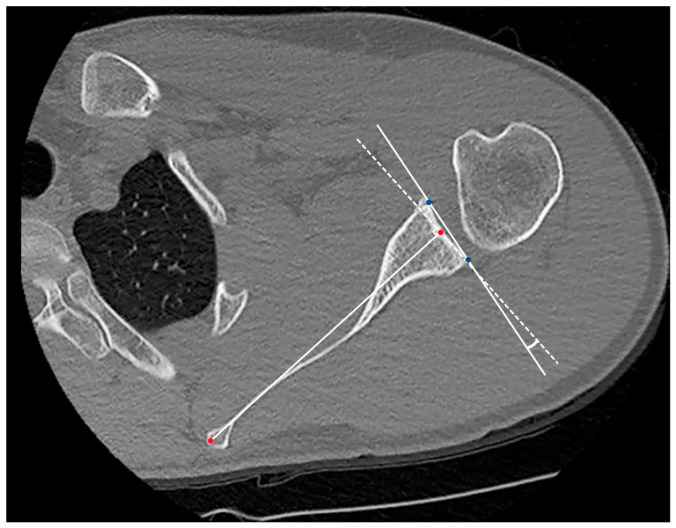
The first axial CT image in which the scapular body was completely visualized was selected for measurement using the Friedman method. The glenoid line (solid line connecting the blue dots) connects the anterior and posterior glenoid margins. The scapular axis (solid line connecting the red dots) extends from the root of the scapular spine to the midpoint of the glenoid fossa. Glenoid version was measured as the angle between the glenoid line and the line perpendicular to the scapular axis (dashed line).

**Figure 4:**
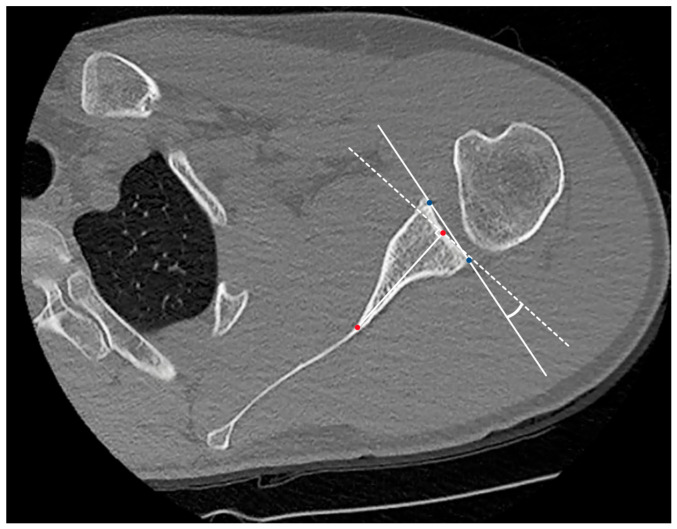
The first axial CT image in which the scapular body was completely visualized was selected for measurement using the Vault method. The glenoid line (solid line connecting the blue dots) connects the anterior and posterior glenoid margins. The vault line (solid line connecting the red dots) extends from the apex of the scapular vault to the midpoint of the glenoid fossa. Glenoid version was measured as the angle between the glenoid line and the line perpendicular to the vault line (dashed line)

The glenoid version was evaluated by two different methods, both defined in the literature and both can be measured on axial CT images. The Friedman method is the traditional method to evaluate the glenoid version [17,18]. As described in the literature, the measurement is performed in axial section shoulder CT, in the first image where the scapula body can be seen completely. Glenoid version using the Friedman method was determined as follows. The angle formed between the glenoid line, connecting the anterior and posterior glenoid margins, and a line perpendicular to the scapular axis extending from the root of the scapular spine to the midpoint of the glenoid fossa was measured (Friedman Angle; FA) (Figure 3). The Vault method, which is a more up-to-date and practical measurement, is also performed in axial section shoulder CT, in the first image where the body of the scapula is fully visible [19]. Glenoid version using the Vault method was determined as follows. The angle formed between the glenoid line, connecting the anterior and posterior glenoid margins, and a line perpendicular to the vault line extending from the apex of the scapular vault to the midpoint of the glenoid fossa was measured (Vault Angle; VA) (Figure 4). Regardless of which method is used, positive values are considered as retroversion and negative values are considered as anteversion [6,18].

All morphometric measurements were performed twice using the digital measurement tools of the Picture Archiving and Communication System (ExtremePACS, Ekstrem Bilgisayar Ltd. Şti., Ankara, Türkiye) by the same senior musculoskeletal radiologist (İSD). The mean of the two measurements was used for the final statistical analysis. All measurements made on direct radiography were performed on standard shoulder radiographs. In the standardization of graphs, several criteria such as “no visible motion,” “greater tubercle on the lateral aspect of proximal humerus,” “humerus head slightly overlapping the glenoid cavity,” and “lesser tubercle located between the greater tubercle and the humerus head” were used [20].

Statistical analyses were performed using IBM SPSS Statistics for Windows, Version 26.0 (IBM Corp., Armonk, NY, USA). Angular measurements, including glenoid version, inclination, and retroversion, were treated as continuous anatomical variables rather than circular directional data. Consequently, they were analyzed using conventional statistical methods. The distribution of continuous variables was assessed using visual methods (histograms and probability plots) and analytical methods (Kolmogorov–Smirnov and Shapiro–Wilk tests). In the case of normally distributed variables, the mean (minimum–maximum) was presented, whereas for non-normally distributed variables, the median (minimum–maximum) was used. Categorical variables were summarized as frequencies and percentages. The associations between glenoid morphometric parameters and fracture characteristics were assessed using Spearman’s rank correlation coefficient. This was due to the primary objective being to assess the strength and direction of the association, rather than assuming a strictly linear relationship. Furthermore, Spearman’s correlation is more robust to outliers and potential deviations from linearity, rendering it well suited to the analysis of biologically variable morphometric measurements. The comparison of categorical variables was performed using either the Chi-square test or Fisher’s exact test, as appropriate. To evaluate whether glenoid morphologic parameters were independently associated with fracture severity after accounting for potential confounding factors, multivariable ordinal logistic regression analyses were performed using Neer and AO/OTA classifications as dependent variables. The following independent variables were entered into the model: glenoid inclination, critical shoulder angle, glenoid version (Friedman angle), vault angle, age, sex, and injury mechanism. Adjusted odds ratios (ORs) with corresponding 95% confidence intervals (95% CIs) were calculated for each predictor. A two-sided *p* value <0.05 was considered statistically significant.

## 3. Results

Of the 113 patients included in the study, 67 (59.3%) were female and 46 (40.7%) were male. Average age was 55.4 years (Range: 19–93 years). Older age was found to be correlated with higher Neer and AO/OTA fracture types (r = 0.176, *p* = 0.031; r = 0.208, *p* = 0.013). Detailed distribution of demographic data of the patients based on Neer and AO/OTA Classifications can be seen in Table 1 and Table 2, respectively.

Across all fracture types, the proportion of female patients was higher than the proportion of male patients. Furthermore, right-sided fractures were observed to occur with a slightly higher frequency than left-sided fractures. However, these differences did not reach statistical significance and should therefore be regarded as descriptive findings within the context of the study population.

While glenoid inclination was found to be correlated with fracture geometry and fragmentation (r = 0.228, *p* = 0.007), no other correlation was found between anatomical parameters and Neer Classification (*p* > 0.05 for each). All anatomical parameters and their relationship with Neer Classification can be seen in Table 3.

Glenoid inclination and glenoid version calculated by Friedman Angle were found to be correlated with the AO/OTA Classification (r = 0.181, *p* = 0.028, and r = −0.207, *p* = 0.014, respectively). On the other hand, no correlation was found between the glenoid version calculated by the vault angle and articular surface involvement (r = −0.113, *p* = 0.116). All anatomical parameters and their relationship with AO/OTA Classification can be seen in Table 4.

### Multivariable Ordinal Logistic Regression Analysis

Because age demonstrated significant univariate correlations with both fracture classification systems, multivariable ordinal logistic regression analyses were subsequently performed to ascertain whether glenoid morphologic parameters were independently associated with fracture severity, with adjustment for potential confounding variables (see Table 5). Following adjustment for age, sex, injury mechanism, critical shoulder angle, glenoid inclination, Friedman angle, and vault angle, no evaluated glenoid morphologic parameters remained independently associated with increasing Neer fracture severity. Glenoid inclination demonstrated a borderline association with higher Neer classification (adjusted OR 1.03, 95% CI 1.00–1.07; *p* = 0.083), whereas CSA, Friedman angle, and vault angle were not independently associated with fracture severity (all *p* > 0.05). Conversely, female sex and injury mechanism were not found to be independent predictors of outcome, although direct trauma demonstrated a borderline association (adjusted OR 2.56, 95% CI 0.91–7.22; *p* = 0.075).

In a similar manner, within the AO/OTA model, age was the solitary independent predictor of increasing fracture severity (adjusted OR 1.03, 95% CI 1.00–1.05; *p* = 0.049). Neither glenoid inclination (adjusted OR 1.01, 95% CI 0.97–1.05; *p* = 0.728), Friedman angle (adjusted OR 0.95, 95% CI 0.87–1.04; *p* = 0.289), vault angle (adjusted OR 1.02, 95% CI 0.94–1.11; *p* = 0.625), CSA (adjusted OR 1.02, 95% CI 0.96–1.09; *p* = 0.439), sex, nor injury mechanism remained independently associated with AO/OTA fracture severity.

## 4. Discussion

A better understanding of the relationship between glenoid morphology and proximal humeral fracture geometry may facilitate a more comprehensive understanding of fracture mechanisms. In the present study, greater GI was associated with more complex fracture patterns according to both the Neer and AO/OTA classifications in the univariate analyses. However, these associations were no longer statistically significant after adjustment for age, sex, and injury mechanism in the multivariable ordinal logistic regression analyses. Likewise, the association between glenoid version and AO/OTA fracture type was not maintained after multivariable adjustment. These findings indicate that GI was not an independent predictor of fracture configuration after accounting for available patient- and injury-related factors, suggesting that glenoid morphology should be interpreted as one of several contributors rather than a standalone determinant of fracture configuration. Nevertheless, the observed univariate associations indicate that glenoid morphology may still play a role in fracture morphology and warrant further investigation in larger prospective studies. A better understanding of glenoid anatomical characteristics may eventually broaden our understanding of fracture mechanisms and support future research into fracture-specific implant technologies and surgical planning. Given the recognized influence of glenoid morphology on other shoulder disorders as evidenced by the extant literature [15,16,18,19,21], further investigation into its relationship with proximal humeral fracture characteristics appears warranted.

Increased GI has been associated with superior rotation of the glenoid, narrowing of the subacromial space, and glenohumeral pathologies such as shoulder instability, rotator cuff tears, and glenohumeral arthritis [15,16,19,21]. In the univariate analyses, GI was associated with both the Neer and AO/OTA fracture classifications, with the highest mean values observed in Neer Type 3 and AO/OTA type 11B fractures (21.4° and 21.4°, respectively). However, this association was no longer statistically significant after adjustment for age, sex, and injury mechanism in the multivariable analyses. To our knowledge, no previous studies have specifically examined the relationship between GI and proximal humeral fracture configuration. One possible explanation is that greater GI may alter glenohumeral joint orientation and load transmission during trauma, potentially predisposing the proximal humerus to more complex fracture patterns. However, this hypothesis remains speculative and requires confirmation through future biomechanical and anatomical studies.

The Friedman Angle is a reliable method for glenoid version assessment [17]. On the other hand, Matsumura et al. stated that scapular body shape has a substantial influence on glenoid version measurements obtained using the Friedman Angle and introduced the Vault Angle to minimize this effect [18]. In the present study, glenoid version measured using the Friedman Angle was associated with the AO/OTA classification in the univariate analyses (*p* = 0.028, r = −0.207). However, this association was no longer statistically significant after adjustment for age, sex, and injury mechanism in the multivariable analyses. In contrast, glenoid version measured using the Vault Angle was not associated with proximal humeral fracture geometry in either the univariate or multivariable analyses. Although these findings do not support glenoid version as an independent predictor of fracture configuration, they raise the possibility that differences between measurement techniques may reflect the influence of scapular morphology rather than glenoid version alone. To our knowledge, no previous studies have specifically examined this relationship, and no established biomechanical mechanism currently explains these observations. Therefore, further biomechanical and clinical investigations are warranted to clarify the potential contributions of glenoid version and scapular morphology to proximal humeral fracture geometry. Almost all previous studies have focused on the relationship between glenoid version and shoulder pathologies or shoulder arthroplasty outcomes [5,11,18,22,23,24]. Additional biomechanical studies are needed to better understand the potential influence of glenoid and scapular body orientation on proximal humeral fracture geometry.

CSA is a strong predictor of degenerative rotator cuff tears and primary glenohumeral arthritis [10,15,25]. Additionally, it has been associated with postoperative range of motion following shoulder arthroplasty [8]. In the present study, however, CSA was not associated with proximal humeral fracture geometry in either the univariate or multivariable analyses. At first glance, this finding appears inconsistent with studies reporting a correlation between CSA and GI [6,15,26]. However, the relationship between CSA and GI remains controversial, as several studies have reported no significant association between these parameters [27,28]. CSA has been linked to lateralization of the deltoid and rotator cuff muscles and increased shear stress across the glenohumeral joint, which may explain its association with degenerative rotator cuff tears [6,26]. In contrast, no established biomechanical relationship between CSA and loading patterns contributing to proximal humeral fracture configuration has been demonstrated, which is consistent with our findings.

In the present study, the observed correlations between radiologic morphometric parameters and fracture classification were statistically significant but weak (approximately |r| ≈ 0.2) in the univariate analyses. Furthermore, these associations were no longer statistically significant after adjustment for age, sex, and injury mechanism in the multivariable ordinal logistic regression analyses. Together, these findings indicate that fracture configuration cannot be explained solely by the evaluated radiologic measurements. Instead, these parameters are likely to represent only one component of a more complex process, with additional anatomical, biomechanical, and injury-related factors contributing to fracture morphology. The weak univariate correlations and the absence of independent associations after multivariable adjustment suggest that the evaluated morphometric parameters account for only a limited proportion of the variability in fracture configuration. Therefore, these findings should be regarded as exploratory and hypothesis-generating rather than as evidence of clinically meaningful predictive relationships. From a clinical perspective, radiologic morphometric measurements should be considered complementary tools for fracture assessment rather than standalone predictors of fracture configuration. Treatment planning should continue to integrate morphometric findings with the overall clinical presentation, injury mechanism, and comprehensive imaging assessment.

Older age was associated with greater fracture complexity, demonstrating significant correlations with fracture classification in the univariate analyses. Furthermore, age remained the only independent predictor of increasing AO/OTA classification after adjustment for sex, injury mechanism, and radiologic morphometric parameters in the multivariable ordinal logistic regression analysis. In contrast, no significant associations were observed between sex, side, injury mechanism, and fracture configuration. These findings are generally consistent with previous reports. A biomechanical study by Majed et al. demonstrated that cortical thinning, which is closely related to aging, is strongly associated with proximal humeral fractures [3]. One methodological consideration should be acknowledged. Although patients with proximal humeral fractures routinely underwent CT before orthopedic consultation in our institution, 39 patients were excluded because their radiographs or CT images were unavailable. This raises the possibility that older patients with relatively simple fracture patterns who did not undergo CT, or frail patients unable to undergo CT, may have been underrepresented. However, the excluded patients had a mean age of 63.3 years (range, 19–82 years), suggesting that exclusions were not limited to elderly or frail individuals but occurred across a broad age range. Nevertheless, the influence of age on proximal humeral fracture configuration should be confirmed in prospective studies with standardized imaging protocols.

In the present study, women accounted for a greater proportion of patients across all fracture classifications (Table 1 and Table 2), consistent with the well-established epidemiology of proximal humeral fractures. Given the relatively advanced mean age of the cohort, this finding is most likely explained by the higher prevalence of postmenopausal osteoporosis and the resulting susceptibility to low-energy fragility fractures among women. Previous population-based studies have similarly demonstrated a higher incidence of proximal humeral fractures in women, particularly in older age groups [29]. Right-sided fractures were also observed slightly more frequently than left-sided fractures. Although this difference was not statistically significant, it may reflect the predominance of right-hand dominance in the general population and the instinctive use of the dominant upper extremity during a fall. However, this observation should be interpreted with caution, as the present study was not designed to evaluate the influence of limb dominance on fracture occurrence or fracture configuration.

Proximal humeral fractures are recognized as multifactorial injuries influenced by a combination of demographic, clinical, and anatomical factors. Numerous studies have identified advanced age, female sex, osteoporosis, reduced bone mineral density, and low-energy falls as the strongest predictors of fracture risk [29,30,31,32,33,34]. In contrast, radiographic investigations have primarily focused on bone quality, humeral morphology, and fracture classification, whereas the potential contribution of native glenoid morphology has received considerably less attention. In the present study, glenoid morphometric parameters demonstrated weak associations with fracture configuration in the univariate analyses; however, these associations were not maintained after adjustment for age, sex, and injury mechanism in the multivariable analyses. These findings suggest that glenoid morphology alone is unlikely to be an independent determinant of fracture configuration but may represent one of several anatomical factors contributing to fracture patterns. Together with the existing literature, our results support the concept that proximal humeral fracture morphology is determined by the complex interaction of patient-specific anatomy, bone quality, injury mechanism, and other biomechanical factors rather than by any single factor alone. In addition to bone morphology and bone quality, loading patterns, shoulder kinematics, muscle function, and injury mechanism may all contribute to fracture occurrence and configuration. Recent evidence has further highlighted the importance of biomechanical characteristics in upper-extremity injury risk, particularly during overhead athletic activities, underscoring the influence of mechanical loading on osseous and periarticular structures [35]. Although these findings may not be directly applicable to the predominantly older population included in the present study, they reinforce the broader concept that individual biomechanical factors should be considered when investigating fracture mechanisms. Future prospective studies integrating radiographic, biomechanical, and functional assessments may provide a more comprehensive understanding of the factors contributing to proximal humeral fracture configuration.

There are several limitations to our study. First, the Walch Classification, which is based on morphological parameters of the glenoid, was not taken into consideration in our study. Since this classification system is associated with glenohumeral arthritis in the literature [36], we chose not to use this classification when performing the morphological analysis. Nonetheless, considering the premise of our study was based on Neer and AO/OTA Classifications, the moderate reliability (both inter and intra) of these classifications is an important limitation. Secondly, the retrospective design of our study and the fact that measurements were conducted on the radiographs taken in the emergency room after an acute scenario are important limitations. Any rotation/incorrect positioning related to the acute pain will affect the measurement. Moreover, although these measurement methods are defined in the literature and often preferred with high reliability, CSA and GI measurements are only based on anteroposterior radiographs in our study. It may be possible to obtain different results with measurements made on 3D-section CT images or MRI images conducted electively. A further limitation of this study is the fact that glenoid morphometric measurements were obtained from two-dimensional CT images that had been standardized, rather than from three-dimensional reconstructed bone models, which constitutes a significant limitation. While this approach is reflective of routine clinical practice and enabled the inclusion of a larger study population, future studies utilizing standardized 3D reconstructions may offer a more detailed characterization of glenoid morphology and its relationship with fracture patterns. Furthermore, our study is a clinical and radiological study examining the effect of anatomical parameters of the glenoid on the proximal humeral fracture geometry. However, different results may be obtained by combining clinical studies with finite element analysis or biomechanical studies.

Finally, it is imperative to acknowledge the several statistical and methodological limitations of the present study. Although multivariable ordinal logistic regression analyses were performed to adjust for age, sex, and injury mechanism, residual confounding cannot be completely excluded. Direct measures of bone quality, such as bone mineral density or osteoporosis severity, were unavailable and therefore could not be incorporated into the analyses. Sex-stratified analyses were also not performed because of limited subgroup sizes. In addition, although statistically significant associations were identified in the univariate analyses, these associations were no longer statistically significant after multivariable adjustment, and the corresponding correlation coefficients were weak. Therefore, the findings should be regarded as exploratory and hypothesis-generating rather than as evidence of clinically meaningful predictive relationships or as standalone tools for fracture risk stratification or clinical decision-making.

## 5. Conclusions

The present study demonstrated weak but statistically significant associations between glenoid anatomical characteristics and proximal humeral fracture configuration in the univariate analyses. Greater GI was associated with more complex fracture patterns, whereas glenoid version was associated with fractures involving the articular surface. However, these associations were no longer statistically significant after adjustment for age, sex, and injury mechanism, suggesting that glenoid morphology is unlikely to represent an independent predictor of fracture configuration.

Although the present findings do not support the clinical use of glenoid morphometric parameters as standalone predictors of fracture patterns, they provide additional insight into the complex anatomical and biomechanical factors contributing to proximal humeral fractures and may serve as a foundation for future hypothesis-driven research. Further prospective clinical, anatomical, biomechanical, and finite element studies incorporating larger cohorts are warranted to confirm these findings and further clarify the potential contribution of glenoid morphology to fracture configuration.

## Figures and Tables

**Table 1:** Demographic profile of the patients and their relationship with Neer Classification.

		Neer Classification				Total (*N* = 113)	*p* (*r*)
		Type 1 (*N* = 15)	Type 2 (*N* = 37)	Type 3 (*N* = 46)	Type 4 (*N* = 15)		
Age		50.1 (23–83)	52.4 (19–89)	59.5 (24–93)	55.9 (21–76)	55.4 (19–93)	0.031 (r = 0.176)
Gender	Female	6 (40%)	22 (59.5%)	30 (65.2%)	9 (60%)	67 (59.3%)	0.411
	Male	9 (60%)	15 (40.5%)	16 (34.8%)	6 (40%)	46 (40.7%)	
Side	Right	5 (33.3%)	19 (51.4%)	26 (56.5%)	8 (53.3%)	58 (51.3%)	0.501
	Left	10 (66.7%)	18 (48.6%)	20 (43.5%)	7 (46.7%)	55 (48.7%)	
Injury Mechanism	Low-energy Fall	12 (80%)	27 (73%)	37 (80.4%)	10 (66.7%)	86 (76.1%)	0.474
	High-energy Fall	1 (6.7%)	5 (13.5%)	1 (2.2%)	1 (6.6%)	8 (7.1%)	
	Direct Trauma	2 (13.3%)	5 (13.5%)	8 (17.4%)	4 (26.7%)	19 (16.8%)	

P: Statistical significance value; r: Spearman’s rho correlation coefficient value. Mean and minimum–maximum range were used for Age variable, and frequency tables were used for nominal variables.

**Table 2:** Demographic profile of the patients and its relationship with AO/OTA Classification.

		AO/OTA Classification			Total (*N* = 113)	*p* (*r*)
		Type 11A (*N* = 51)	Type 11B (*N* = 48)	Type 11C (*N* = 14)		
Age		51.4 (19–89)	58.2 (21–93)	60.6 (37–90)	55.4 (19–93)	0.013 (r = 0.208)
Gender	Female	26 (51%)	32 (66.7%)	9 (59.3%)	67 (59.3%)	0.271
	Male	25 (49%)	16 (33.3%)	5 (35.7%)	46 (40.7%)	
Side	Right	26 (51%)	24 (50%)	8 (57.1%)	58 (51.3%)	0.928
	Left	25 (49%)	24 (50%)	6 (42.9%)	55 (48.7%)	
Injury Mechanism	Low-energy Fall	39 (76.5%)	35 (72.9%)	12 (85.7%)	86 (76.1%)	0.349
	High-energy Fall	6 (11.8%)	2 (4.2%)	0	8 (7.1%)	
	Direct Trauma	6 (11.8%)	11 (22.9%)	2 (14.3%)	19 (16.8%)	

P: Statistical significance value; r: Spearman’s rho correlation coefficient value. Mean and minimum–maximum range were used for Age variable, and frequency tables were used for nominal variables.

**Table 3:** Relationship between glenoid morphology and Neer Classification.

	Neer Classification				*p* (*r*)
	Type 1 (*N* = 15)	Type 2 (*N* = 37)	Type 3 (*N* = 46)	Type 4 (*N* = 15)	
CSA (degree)	42.3 (34.6–57.6)	42.5 (24.8–58.4)	44 (26.8–63)	41.5 (31.9–51.5)	0.361 (0.034)
GI (degree)	16 (3.5–32.7)	17.3 (−10.3–60.4)	21.4 (1.5–55)	19.9 (0.2–38.7)	0.007 (0.228)
Glenoid Version					
Friedman Angle (degree)	−2.4 (−15–9.7)	0.678 (−10.5–12.9)	−1.6 (−18.3–8.9)	1.7 (−7.9–27.3)	0.461 (0.009)
Vault Angle (degree)	4.4 (−13–15.5)	8.1 (−5.8–18.2)	4.6 (−25.5–16)	6.3 (−2.5–27)	0.437 (−0.015)

CSA: Critical shoulder Angle; GI: glenoid inclination; P: Statistical significance value; r: Spearman’s rho correlation coefficient value. Median and minimum–maximum range were used for Vault Angle and Alpha Angle variables, whereas mean and minimum–maximum range were used for all the remaining variables.

**Table 4:** Relationship between glenoid morphology and AO/OTA Classification.

	AO/OTA Classification			*p* (*r*)
	Type 11A (*N* = 51)	Type 11B (*N* = 48)	Type 11C (*N* = 14)	
Critical Shoulder Angle (degree)	42.1 (24.8–58.4)	43.7 (26.8–63)	43.6 (38.2–51.5)	0.114 (0.114)
Glenoid Inclination (degree)	17.1 (−10.3–60.4)	21.4 (1.5–55)	18.5 (0.2–38.7)	0.028 (0.181)
Glenoid Version				
Friedman Angle (degree)	0.9 (−15–12.9)	−2.2 (−18.3–27.3)	−0.1 (−7.9–10.1)	0.014 (−0.207)
Vault Angle (degree)	7.9 (−13–18.2)	4.4 (−25.5–27)	7.6 (−2.5–14.6)	0.116 (−0.113)

P: Statistical significance value; r: Spearman’s rho correlation coefficient value. Median and minimum–maximum range were used for Vault Angle and Alpha Angle variables, whereas mean and minimum–maximum range were used for all the remaining variables.

**Table 5:** Multivariable ordinal logistic regression analysis of factors associated with increasing fracture severity according to the Neer and AO/OTA classifications.

Predictor	Neer Adjusted OR (95% CI)	*p*	AO/OTA Adjusted OR (95% CI)	*p*
Age (per year)	1.02 (1.00–1.05)	0.071	1.03 (1.00–1.05)	0.049
Female Sex	1.49 (0.65–3.40)	0.344	1.04 (0.44–2.47)	0.920
High-energy Fall *	0.90 (0.23–3.55)	0.884	0.38 (0.07–2.16)	0.277
Direct Trauma *	2.56 (0.91–7.22)	0.075	2.31 (0.81–6.58)	0.116
Glenoid Inclination (per degree)	1.03 (1.00–1.07)	0.083	1.01 (0.97–1.05)	0.728
Critical Shoulder Angle (per degree)	0.99 (0.93–1.05)	0.682	1.02 (0.96–1.09)	0.439
Glenoid Version				
Friedman Angle (per degree)	1.07 (0.98–1.17)	0.112	0.95 (0.87–1.04)	0.289
Vault Angle (per degree)	0.98 (0.91–1.06)	0.590	1.02 (0.94–1.11)	0.625

OR: odds ratio; CI: confidence interval. * Reference category: low-energy fall.

## Data Availability

All data have been deposited in a private repository but are available from the corresponding author on reasonable request.

## Abbreviations

The following abbreviations are used in this manuscript:

CT	Computed tomography
CSA	Critical shoulder angle
GI	Glenoid inclination
FA	Friedman Angle
VA	Vault Angle
